# Annotations

**Published:** 1899-03-25

**Authors:** 


### Annotations.

### Ethics and Advertising.

We have received a copy of a Paris paper called the *English and American Gazette*, the leading article in which is devoted to a lament over the English medical profession and the distressful position in which its members find themselves from submitting to the domination of "that terrible bogey of professional etiquette." We are told that "the British medical man in practice abroad is going to the wall," and all apparently because of etiquette. Medical etiquette, no doubt, means very different things in different minds. Those who know most on the subject know that there is no such thing, and that what seems to some so "terrible" a "bogey" is but the application to the affairs of daily professional life of ordinary rules of gentlemanly conduct. That such rules tend to prevent self-advertisement and to place beyond the pale those who would take advantage of their fellows is true enough, but we fail to see why what in one walk of life would be considered proper and correct should in another be stigmatised as obedience to mere medical etiquette, and be spoken of, as it often is, as a something not far removed from medical trades unionism. The fact is, that all this talk among non-professional people about the tyranny of professional etiquette arises from the idea, so common among the public, that they are our employers, and that we ought to mould our conduct according to their desires. When they ask us to do things that no gentleman would for a moment think of doing, and when we refuse—refuse, for example, to see another man's patient behind his back, or to go through the farce of a consultation with a homœopath—it does not seem to occur to people that the fact that such actions would be dishonourable is any excuse for our declining to do them. So they put what they would call our perversity down to "medical etiquette," a thing which they declare they never could understand—as, indeed, we are fain to believe that some of them never could. We cannot, then, sympathise in the least with the editor of the *English and American Gazette* in his lament, or join him in his assertion that a medical man is "very wrong, and more, is absolutely criminal, if he allows professional etiquette to carry him so far as to refuse his name's free admission to the columns of a newspaper," with the object of "telling the public, who have no other means of knowing, that there exists in such-and-such a town a medical man . . . who can be called in case of illness." We have an old-fashioned belief that any such advertisement is bad form, and in this we fancy that the mass of the profession will agree. Not all, however; for if we turn over the pages of this very paper which writes so indignantly about the evils of "professional etiquette" one finds plenty to show that its editor need not have been so urgent in bemoaning the exceeding virtue of the profession—a virtue not altogether inaccessible to assault, if we may judge by the long list of medical men whose names appear in its advertisement columns. It is an ingenious device, no doubt, instead of putting in a bald advertisement stating that Dr. A. practices in one place and Dr. B. in another, to advertise a selection from among the doctors practising in each of the principal health resorts of Europe, and then to head the list with the legend "Continental Anglo-American Medical Society," but it does not seem to us to make the advertisement any the less objectionable. It is high time that this society made up its mind what its function is to be. If it is an ordinary professional association working on professional lines, surely it ought to be able to protect itself from such a misuse of its list of members; while if, as from the appearance of this list week after week would seem to be the case, it is merely a mutual association for advertising purposes, we think that the Royal College of Physicians might well bring a little gentle pressure to bear on those of its Fellows who join in such proceedings.

### The Workmen's Houses Bill.

We always look with supreme suspicion upon attempts to interfere with the ordinary course of trade by Government subvention, and when we find a Bill brought forward with the definite object of enabling local bodies to enter upon a business which has already been undertaken by private persons with a considerable amount of success, namely, the making of advances to householders for the purchase of their dwellings, we feel inclined to inquire not merely whether the business to be embarked in is likely to be successful, but whether, if successful, it will be for the good of the community. We do not, however, now propose to enter on the question whether the Workmen's Houses Bill is likely to be a success as a matter of economics, but we feel bound to say that any large extension of the plan of working people becoming owners of their dwellings should be regarded with great suspicion from a sanitary and public health point of view. Every new development of municipal sanitation makes it the more essential that the sanitary authority should be able to get at a solvent and responsible

ANNOTATIONS—*continued.*

owner for every house within its area; and we ask, Is it for the public good and for the sanitary advantage of the people at large that house property in towns should be held in small blocks, or, still more, in single tenements by working people whose capital is of the smallest, and who from the circumstances of the case must look with grudging eye upon every penny expended in sanitary improvements? To this there can be but one answer. It is the universal experience of sanitary authorities that house property held by impecunious owners is most difficult to deal with. The workman living in his own house is almost always an obstructive. He "puts up with" bad drains, he "puts up with" damp walls and imperfect plumbing rather than spend a penny in putting things right, whereas if he were an ordinary rent-paying tenant he would set the whole sanitary machinery of his town in operation (at the town's expense) to make his landlord put things in order. But the poor widow drawing her mite from the house in which her husband has invested his savings is an almost more unsatisfactory owner. She lets the house at such rent as she can get, but she has not a penny at her back, and any sanitary repairs which may be necessary are done in the cheapest and most perfunctory manner. It is constantly found to be the case that house property owned by poor people tends quickly to become a centre of insanitation. It is easy to say that this need not be the case, and of course it need not. All we have to do when the widow or the poor children of the thrifty workman cannot keep their property in good order is to sell them up. But this is a hardship and a cruelty, to avoid doing which elected bodies who have to answer to the public will go a long way, and the fact remains that so far as sanitation is concerned the single house belonging to a poor owner stands at a great disadvantage compared with the street or the estate owned by some capitalist or public company which can be forced to maintain the property in good sanitary repair. Under active sanitary supervision the capitalist is the best landlord, and this we say not from any special sympathy with him. It has been said that limited companies have neither souls to be saved nor bodies to be kicked, and this is taken as the explanation of their many misdeeds. On the other hand, no one cares about the feelings of a company, and it can always be made to "pay up," which, after all, so far as the sanitary authority is concerned, is the great thing.

### Bacteriology in France.

It is fitting that the native country of Pasteur should take a leading position in the development of the great science which has sprung out of his researches; and a member of the Chamber of Deputies, M. Emile Dubois, has submitted to the Chamber a project of law for the establishment in every department, at the public cost, of one or more bacteriological laboratories, for the conduct of researches with a view to the prevention of contagious diseases in general, and of tuberculosis in particular. The state of business renders it probable that a considerable time must elapse before the proposal can be reported upon and discussed; but the project provides that the public laboratories to be established shall be at the disposal of all medical and veterinary practitioners, and of all *sages-femmes*; and that all investigations shall be conducted free of expense to applicants, except for the costs of carriage and correspondence. In the interests alike of public health and of civilisation, it is to be hoped that M. Dubois will succeed in obtaining the enactment which he desires, and will thus give the world occasion to include bacteriology among the other things which "they do better in France."

### Superannuation.

Those who, acting in all good faith, devised the Poor-Law Superannuation Act (1896), hoping thereby to give to a large class of valuable public servants a certainty of having some small pension as a comfort and assistance in their old age, must be rather disappointed at the way in which the working of that Act is turning out in practice, for the Guardians appear to have quickly discovered that there is a loophole by aid of which they may escape the irksome necessity of paying these annuities at all. What is becoming evident is that if the Guardians can but get rid of their old servants before the age at which they become eligible for pensions all claim upon them is removed, and what is also being made very evident is that such Boards of Guardians as will take advantage of this means of relieving the rates will certainly take care not to engage afresh anyone who is approaching a pensionable age. On nurses and attendants and porters, and other servants who can be dismissed, the hardship is very great; for, although probably no Board of Guardians has yet been found so callous as openly to dismiss a servant because he was nearing the age at which a pension might be due, it is obvious enough that as that age approaches such a servant becomes the absolute slave of his masters and dare not rebel, whatever burden may be put upon him. His "golden fetters" are hard indeed, for he knows that if he resigns he certainly will not get another place, and he knows also that if he is not in Poor Law employ at the time he becomes old enough to be eligible for a pension he will not get one, however many years he may have suffered the statutory deduction from his wages. It may be said that medical officers cannot be dismissed; but they can be made uncomfortable, and as years go on and as the payment of pensions under the Act becomes a larger and more irksome item of Poor Law expenditure, taken as they are out of the funds of the individual union, we may be pretty sure that the possibility of getting rid of old officers before their claims become due will often lead, indirectly, perhaps, but none the less effectually, to indignities being heaped upon them which they would have escaped had their pensions been drawn, as they ought to have been, from a common fund, instead of having to be paid by the particular union in whose service they may happen to be at the moment when the pension becomes due. The *Lancet* has indeed specially drawn attention to the fact that medical officers who do not reside within their districts have no security of tenure, and are practically at the mercy of the Guardians, it being stated that in several instances of this nature medical officers approaching a pensionable age have been displaced by younger men, although having a blameless record, and have thus been deprived of the pension which it was the intention of the Act to give them.

